# Computed Tomography Angiography in Peripheral Arterial Disease: Comparison of Three Image Acquisition Techniques to Optimize Vascular Enhancement—Randomized Controlled Trial

**DOI:** 10.3389/fcvm.2020.00068

**Published:** 2020-04-28

**Authors:** David C. Rotzinger, Tri-Linh Lu, Aida Kawkabani, Pedro-Manuel Marques-Vidal, Gianluca Fetz, Salah D. Qanadli

**Affiliations:** ^1^Cardiothoracic and Vascular Division, Department of Diagnostic and Interventional Radiology, Lausanne University Hospital, Lausanne, Switzerland; ^2^Faculty of Biology and Medicine, University of Lausanne, Lausanne, Switzerland; ^3^Groupement Hospitalier de L'Ouest Lémanique, Nyon, Switzerland; ^4^Department of Internal Medicine, Lausanne University Hospital, Lausanne, Switzerland; ^5^Clinica Sant'Anna, Sorengo, Switzerland

**Keywords:** peripheral arterial disease, computed tomography angiography, image quality, contrast media, trial

## Abstract

**Objectives:** To prospectively compare three image acquisition techniques in lower extremity CT angiography: the “standard” anterograde technique (SA), the adaptive anterograde technique (AA), and the retrograde acquisition technique (RA).

**Materials and Methods:** Sixty consecutive patients were prospectively enrolled and randomized into three acquisition groups: 20 patients were evaluated with SA, 20 with AA as described by Qanadli et al., and 20 with caudocranial acquisition from the feet to the abdominal aorta (RA). Quantitative image quality was assessed by measuring the intraluminal attenuation at different levels of interest, with a total of 536 levels. Qualitative image quality was assessed by two radiologists in consensus using a Likert scale to rate the arterial enhancement and venous return. For each patient and limb, the presence of occlusive or aneurysmal disease was documented.

**Results:** In quantitative analysis, RA showed lower attenuation values than SA and AA (*p* < 0.01). AA showed the highest and most homogeneous attenuation along the arterial tree. In qualitative analysis, AA had the lowest rate of non-diagnostic vascular segments (3.9%) compared to SA and RA (4.7 and 13.1%, respectively, *p* < 0.01). The influence of venous return was significantly different among the different techniques; venous contamination was particularly prevalent at the aortic level with RA (9.4% of patients, 0% with SA and AA, *p* < 0.01). The presence of stenosis or occlusion had no significant influence on the attenuation values across all levels and acquisition techniques. Conversely, the presence of aneurysmal disease had a significant effect on the luminal attenuation in AA (higher attenuation) and RA (lower attenuation) at the iliac (*p* = 0.03 and 0.04, respectively) and femoral levels (*p* = 0.02 and <0.01, respectively).

**Conclusion:** Considering both quantitative and qualitative analysis, AA performed better than SA and RA, providing the highest percentage of optimal vascular enhancement. AA should be recommended as the technique of choice, specifically in the presence of aneurysmal disease. Alternatively, SA can be useful in case of renal failure, as the test bolus is unnecessary. Finally, the increasing availability of fast CT systems will likely overcome the limitations of RA.

## Introduction

During the last decade, there have been notable technological advances in CT angiography (CTA) techniques. CTA is fast and accurate for the evaluation of peripheral arterial disease (PAD), and provides a diagnostic performance equal to digital subtracted angiography (DSA) (1–8).

Recent multidetector CT (MDCT) technology (64-MDCT and more) allows submillimeter resolution, mandatory for appropriate small vessel assessment. With the increasing speed of acquisition, the entire abdominal aorta and the arterial system of the lower limbs can be sampled within seconds. That being said, such fast CT systems may “outrun” the physiologic progress of the contrast medium bolus, which can be avoided by willingly slowing down the CT system. On the other hand, if the acquisition time is too long, venous return may spoil the quality of the examination. Consequently, it is mandatory to tailor the acquisition to the type of CT system used, the pathology, and the patients' condition. For example, the presence of stenosis, occlusion or aneurysm may slow the arterial flow down (9). Elderly patients, as well as patients with cardiac disease, may also have a slow flow. Different CTA acquisition protocols have been reported to overcome these limitations (4, 10, 11). Fleischmann et al. (10) recommended a standard anterograde method adapted to the speed of the CT system: a slow acquisition (30 mm/s) protocol lasting 40 s with a start when the abdominal aorta is enhanced or a fast acquisition (45–65 mm/s) protocol lasting 20 s with the acquisition start delayed by 20 s. Another technique proposed by Qanadli et al. (11) is based on the measurement of the time needed for the bolus to travel from the abdominal aorta to the popliteal arteries. The acquisition parameters are then individually adapted to fit the patient's circulation as close as possible. This method is said to be adaptive because it is tailored to each patient.

Contrary to the two latter techniques, one can acquire images in a retrograde manner. The 64-MDCT and later generations allow speedy acquisition times (<4 s). With such a high-speed acquisition, it is possible to sample the whole volume of interest from the feet to the abdominal aorta with a single intravenous contrast medium injection, starting when the optimal enhancement is reached in the below-the-knee arterial tree. That could be particularly useful in patients with critical ischemia, where the vessels below the knees may be challenging to examine.

The purpose of this study was to prospectively compare standard anterograde (SA) acquisition to the adaptive acquisition (AA) and retrograde acquisition (RA) techniques, concerning vascular enhancement, homogeneity of enhancement, and the presence of venous return, in patients referred to lower extremity CTA for occlusive or aneurysmal disease.

## Materials and Methods

### Study Population

Sixty consecutive patients were prospectively enrolled in the study between April 2009 and November 2010, 33.3% (15/45) women, mean age 67 years (range 51–89). The inclusion criteria were as follows: age ≥ 18 years, referred to clinically indicated lower extremity CTA for PAD or aneurysmal disease. Exclusion criteria: known hypersensitivity to iodinated contrast media, kidney failure with creatinine clearance lower than 30 mL/min, or denial to participate.

Demographics (age, sex, and body weight), cardiovascular risk factors (diabetes, active smoking, hypercholesterolemia, hypertension, and obesity) and the presence of heart failure (ejection fraction <55% demonstrated with echocardiography) are reported in Table 1.

**Table 1:** Demographic details and characteristics of patients by groups of acquisition.

	**SA**	**AA**	**RA**	
Age [yrs]	65.3 +/– 9.5	70.6 +/– 10.4	65.1 +/– 8.4	*P* = 0.12
Sex ratio [M/W]	17/3	15/5	13/7	*P* = 0.34
Weight [kg]	74.7 +/– 19.0	75.5 +/– 13.9	76.1 +/– 20.4	*P* = 0.97
Symptomatic limbs	27/39^*^	23/40	25/40	*P* = 0.56
Acute ischemia	0/20	1/20	0/20	*P* = 0.33
Stage II claudication	14/20	12/20	17/20	
Stage III-IV claudication	6/20	5/20	2/20	
Asymptomatic (non-PAD)	0/20	2/20	1/20	
Past history of by-pass procedures	7/20	4/20	4/20	*P* = 0.45
Limbs with significant stenosis	33/39^*^	34/40	31/40	*P* = 0.61
Limbs with aneurysms	3/39^*^	6/40	2/40	*P* = 0.28
Heart failure	1/20	2/20	0/20	*P* = 0.67
Diabetes	7/20	4/20	4/20	*P* = 0.45
Smoking	10/20	7/20	7/20	*P* = 0.54
Hypercholesterolemia	12/20	11/20	14/20	*P* = 0.61
Hypertension	15/20	12/20	14/20	*P* = 0.58
Obesity	3/20	1/20	1/20	*P* = 0.78
Aortic aneurysm	3/20	5/20	1/20	*P* = 0.39

*N = 60*.

*SA, standard acquisition technique; AA, adaptive acquisition technique; RA, retrograde acquisition technique; PAD, peripheral arterial disease*.

* *one amputated limb*.

Forty-three patients had stage II PAD according to the Fontaine classification, 13 patients had stage III-IV disease, one patient had acute ischemia, and three patients had aneurysmal disease. Before the examination, each patient was randomly assigned to an acquisition technique using the sequentially numbered opaque sealed envelopes method: 20 patients were evaluated with SA acquisition, 20 with AA, and 20 with RA.

Written informed consent was obtained from every patient participating and the Ethics Committee of the Canton de Vaud approved the study.

### CT Angiography Protocols

All examinations were performed on a 64-MDCT system (LightSpeed VCT, GE Healthcare, Milwaukee, WI, USA). Patients were positioned supine, feet first. The beam collimation geometry was 64 × 0.625 mm, the tube potential 120 kVp, with automatic tube current modulation enabled (100–330 mA). Acquisition covered a volume starting from the T12 level to the feet. Images were reconstructed with a slice thickness of 1.25 mm, using the standard kernel.

In the SA group, images were acquired in the craniocaudal direction after the injection of 100 mL iodinated contrast medium (Accupaque 300, GE Healthcare, Oslo, Norway) into a right brachial vein at a rate of 4 mL/s, followed by 40 mL of saline flush. The acquisition time was fixed at 40 s for every patient. The acquisition was triggered when the abdominal aorta (T12 level) reached an attenuation of 200 Hounsfield units (HU). The rotation time was 0.5 s, and the pitch (always ≤ 1) was adapted to target an acquisition time of 40 s. This method, quoted as the standard anterograde method, is based on techniques previously described by Fleischmann et al. (10).

In the AA group, a test bolus of 30 mL contrast medium (Accupaque 300) was injected into a right brachial vein, then two series of dynamic acquisitions were performed at low radiation dose (120 kVp, 20 mA) at the level of the T12 vertebra (abdominal aorta) and just below the knee (popliteal arteries), respectively. The sampling of the abdominal aorta started 20 s following the beginning of the intravenous injection and lasted for 10 s, whereas the sampling of the popliteal arteries begun immediately after the first one and lasted 30 s. Subsequently, time to the maximal attenuation in the abdominal aorta (T1) and popliteal arteries (T2) was calculated from the time-density curves. In case of discrepancy between the circulation time of both popliteal arteries, the T2 value of the symptomatic side was used. The aortopopliteal transit time was then computed as (T2-T1) (11), and served to adjust the rotation time and the pitch, targeting an acquisition time equal or almost equal to the aortopoplietal transit time. Finally, 100 mL of contrast medium was injected at a rate of 4 mL/s, followed by 40 mL of saline flush. The acquisition was started manually when the abdominal aorta (T12 level) reached 200 HU; the direction of acquisition was craniocaudal.

In the RA group, the direction of acquisition was caudocranial, following 100 mL of contrast medium (Accupaque 300) injected into a right brachial vein at a rate of 4 mL/s, followed by 40 mL of saline flush. The retrograde acquisition was performed at the highest possible speed (rotation time of 0.5 s, pitch 1.375) with a start when the popliteal arteries reached 100 HU.

### Qualitative and Quantitative Analysis

**Figure 1:**
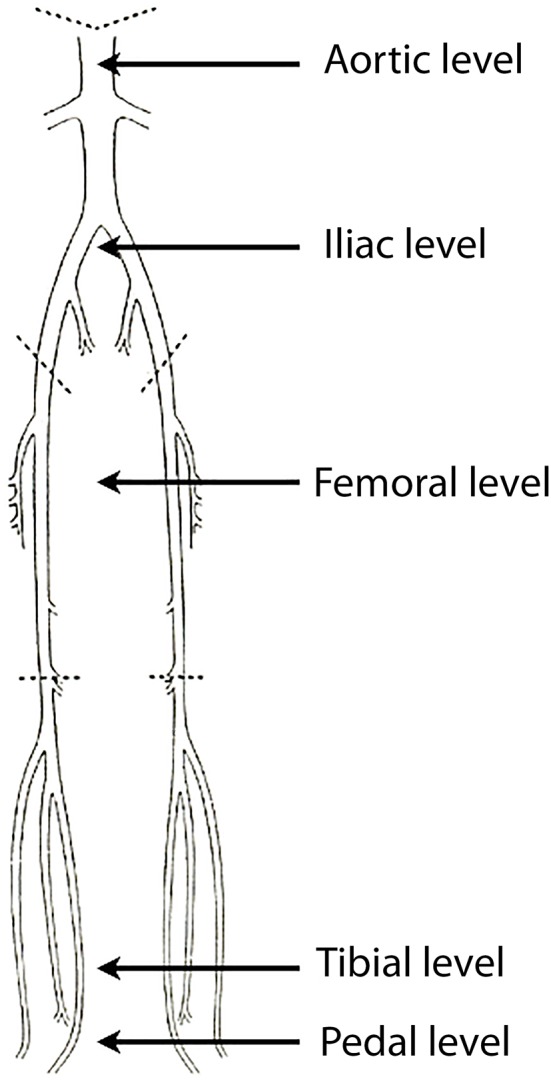
Graphical representation of the arterial supply of the lower limbs. Intraluminal CT values were measured at five levels of interest (black arrows): aortic level, iliac level, femoral level, tibial level, pedal level.

Two radiologists reviewed the CTAs in consensus on an Advantage Window workstation (version 4.3, GE Healthcare, Buc, France). The arterial intraluminal attenuation was measured on axial slices at five different levels of interest (Figure 1), pre-specified as follows: abdominal aorta at the level of T12 (1, aortic level); common iliac arteries 3 cm below the aortic bifurcation (2, iliac level); superficial femoral arteries at mid-distance between the femoral bifurcation and the ostium of the anterior tibial artery (3, femoral level); posterior tibial arteries 3 cm above the medial malleolus (4, tibial level); plantar arch at the level of the cuboid bone (5, pedal level). Every limb (right and left-sided) was analyzed separately. The attenuation value was then measured by placing a circular region of interest of 0.4 cm^2^ at the two first levels, of 0.1 cm^2^ at the third and the fourth levels, and of < 0.1 cm^2^ at the fifth level. In the case of an occluded vessel, measurements were obtained in a collateral vessel larger than >1 mm or another vessel for tibial and pedal levels.

For each patient and each side, the presence of relevant stenoses (>50% reduction of lumen diameter) and vessel ectasia or aneurysm (aortic and/or peripheral, >30% of the normal vessel diameter) was also reported. The results were stratified likewise.

In order to assess the homogeneity of vascular enhancement along the volume of interest, the relative attenuation difference between two adjacent levels of interest (a and b) along the z-axis [D_(za, zb)_] was computed as follows:

$$\begin{array}{l} D_{\left(\text{za},\text{zb}\right)}=\left[A_{\text{za}}-A_{\text{zb}}\right]/\left[\left(A_{\text{za}}+A_{\text{zb}}\right)/2\right]\times 100 \end{array}$$

Where A_za_ is the attenuation value measured at one level (za) and A_zb_ is the attenuation value measured at the following level in the craniocaudal direction (zb). Mean values were computed for each acquisition technique.

Qualitative assessment of the arterial attenuation was also performed on axial slices, maximum intensity projection (MIP) and volume rendering (VR) reconstructions using a 3-point Likert scale: 0, optimal enhancement; 1, non-optimal enhancement but sufficient to make a diagnosis; 2, non-diagnostic image quality.

Moreover, the impact of enhanced veins (on axial, MIP, and VR reconstructions) was evaluated on a 3-point Likert scale: 0, no venous return; 1, venous return not affecting the interpretation; 2, venous return affecting the interpretation.

### Statistical Analysis

Statistical analyses were conducted using Stata v. 9.2 software (College Station, TX, USA). Data are presented in terms of mean +/– standard deviation (SD) for quantitative variables or the number of patients and proportions (percentage) for qualitative variables.

The population samples in each acquisition group are compared in terms of demographic characteristics and risk factors with a standard Chi-test of independence for high expected frequencies and with Yates correction otherwise.

The comparison of quantitative variables between the three groups was performed with an ANOVA test and a Tukey's honest significance test. Qualitative variables are also compared (Chi-test).

For each group and at each level, the impact of relevant stenoses and aneurysms on contrast medium attenuation was evaluated (*t*-test). *P*-values ≤ 0.05 were considered statistically significant.

## Results

There was no significant difference between groups in terms of age, sex ratio, body weight, symptoms, cardiovascular status, and risk factors (Table 1).

Among all the patients, one had a left amputated limb. Thus, 119 limbs and a total of 536 levels were evaluated.

**Figure 2:**
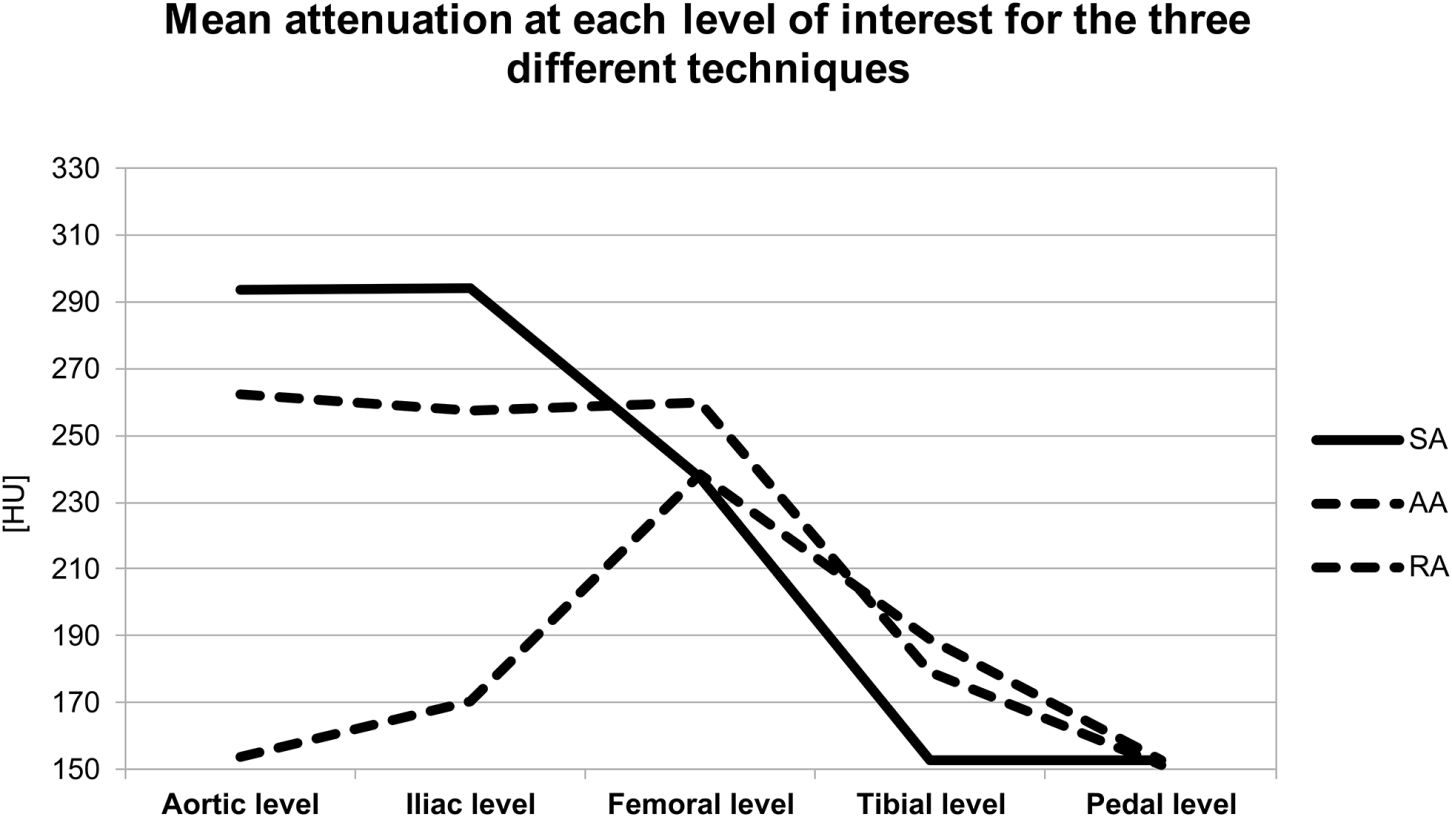
Graph represents the mean attenuation in HU at each level of interest for the three different acquisition techniques. HU, Hounsfield units; SA, standard acquisition technique; AA, adaptive acquisition technique; RA, retrograde acquisition technique.

The level-based mean attenuation for each acquisition technique is presented in Table 2 and Figure 2. The attenuation provided by SA peaked at the aortic level and gradually decreased along the arterial tree. AA provided the most consistent attenuation from the aortic to the superficial femoral artery level, with a decrease at more distal levels. RA provided lower attenuation values at proximal and distal levels, with a peak in the superficial femoral artery.

**Table 2:** Mean attenuation at each level of interest [HU] +/– SD in the whole population.

		**Aorta**	**Iliac artery**	**Superficial femoral artery**	**Tibial artery**	**Pedal artery**
	Right		299.5 +/– 72.4	236.3 +/– 67.9	151.1 +/– 82.3	157.9 +/– 85.1
SA		293.5 +/– 66.3				
	Left		288.9 +/– 75.3	238.0 +/– 59.2	154.3 +/– 85.0	147.6 +/– 73.9
	Right		259.7 +/– 86.5	258.2 +/– 77.8	179.8 +/– 70.8	158.1 +/– 67.1
AA		262.4 +/– 80.4				
	Left		255.4 +/– 84.8	262.0 +/– 82.4	178.7 +/– 77.7	144.9 +/– 54.2
	Right		174.2 +/– 77.6	238.0 +/– 89.1	192.1 +/– 94.4	159.9 +/– 58.0
RA		153.9 +/– 62.3				
	Left		166.0 +/– 70.8	238.3 +/– 85.2	185.7 +/– 84.1	145.7 +/– 69.0

*N = 536 levels*.

*HU, Hounsfield units; SD, standard deviation; SA, standard acquisition technique; AA, adaptive acquisition technique; RA, retrograde acquisition technique*.

The segmental analysis revealed significant differences between the three acquisition techniques for the aortic level (*p* < 0.01). Tukey's *post-hoc* test demonstrated that the RA group had significantly lower attenuation values than the two other groups (*p* < 0.01). At the iliac level, there was also a significant difference between the three techniques (*p* < 0.01). *Post-hoc* testing showed that RA remained significantly different from the two other techniques (*p* < 0.01) with lower attenuation values. There was no significant difference between the three techniques for the femoral level (*p* = 0.20), for the tibial (*p* = 0.13) or plantar level (*p* = 0.99).

When stratified into segments with at least one significant stenosis vs. without stenosis, the results showed no difference for the four distal vascular segments across the three acquisition techniques (Table 3).

**Table 3:** Mean attenuation [HU] +/– SD and univariate comparison at the four distal vascular segments stratified by the presence or absence of stenosis.

		**Iliac artery**		**Superficial femoral artery**		**Tibial artery**		**Pedal artery**	
	Stenosis	286.7 +/– 69.4		237.4 +/– 73.3		152.6 +/– 77.4		157.1 +/– 83.1	
SA			*P* = 0.08		*P* = 0.45		*P* = 0.50		*P* = 0.24
	No stenosis	329.3 +/– 85.6		239.9 +/– 62.0		153.0 +/– 110.4		133.6 +/– 57.1	
	Stenosis	257.4 +/– 86.2		262.3 +/– 88.7		179.3 +/– 71.8		148.2 +/– 61.5	
AA			*P* = 0.49		*P* = 0.36		*P* = 0.50		*P* = 0.21
	No stenosis	258.5 +/– 82.4		247.6 +/– 74.1		179.2 +/– 89.3		170.3 +/– 56.2	
	Stenosis	164.6 +/– 66.3		236.1 +/– 96.8		179.9 +/– 85.5		154.1 +/– 66.6	
RA			*P* = 0.19		*P* = 0.50		*P* = 0.12		*P* = 0.40
	No stenosis	189.1 +/– 96.5		245.3 +/– 80.4		219.7 +/– 95.8		148.2 +/– 53.3	

*N = 476 levels*.

*HU, Hounsfield units; SD, standard deviation; SA, standard acquisition technique; AA, adaptive acquisition technique; RA, retrograde acquisition technique*.

As for the presence of aneurysms, five patients had a peripheral aneurysm on the left side; one patient had a peripheral aneurysm on the right side; six patients had an isolated aortic aneurysm; one patient had an aortic aneurysm with an aneurysm on each limb; one patient had an aortic aneurysm with an aneurysm on the left limb; one patient had an aortic aneurysm with an aneurysm on the right limb. The comparison between limbs with and without aneurysm (aortic and/or peripheral) showed a significant intraluminal attenuation difference at the iliac and femoral levels for AA and RA (AA: *p* = 0.03 and 0.02, respectively; RA: *p* = 0.04 and <0.01, respectively). Note, however that AA provided a higher attenuation at the iliac and femoral levels when an aneurysm was present, unlike RA. Further details are presented in Table 4.

**Table 4:** Mean attenuation [HU] +/– SD at the four distal vascular segments stratified by the presence or absence of aneurysm.

		**Iliac artery**	**Superficial femoral artery**	**Tibial artery**	**Pedal artery**
	Aneurysm	272.5 +/– 109.6	245.5 +/– 95.1	215.5 +/– 137.9	230.5 +/– 154.9
SA					
	No aneurysm	295.3 +/– 72.7	237.4 +/– 70.4	145.3 +/– 82.7	144.7 +/– 77.4
	Aneurysm	316.3 +/– 89.6	308.8 +/– 82.6	168.0 +/– 46.1	130.7 +/– 43.5
AA					
	No aneurysm	247.2 +/– 80.7	251.5 +/– 84.7	181.2 +/– 77.5	155.2 +/– 62.9
	Aneurysm	88.5 +/– 9.2	94.5 +/– 27.7	151.5 +/– 48.8	147.0 +/– 24.0
RA					
	No aneurysm	174.4 +/– 72.9	245.7 +/– 89.0	190.8 +/– 89.8	153.1 +/– 64.8

*N = 476 levels*.

*HU, Hounsfield units; SD, standard deviation; SA, standard acquisition technique; AA, adaptive acquisition technique; RA, retrograde acquisition technique*.

The relative difference of attenuation [D_(za, zb)_] was 10.9 ± 10.8 for SA, 10.4 ± 9.7 for AA, and 15.8 ± 14.9 for RA (*p* < 0.01). Tukey's test showed a significant difference between the RA group and the two other groups (SA and AA) (*p* < 0.01). AA provided the most homogenous enhancement along the acquisition volume even if the difference between AA and SA was not significant.

**Figure 3:**
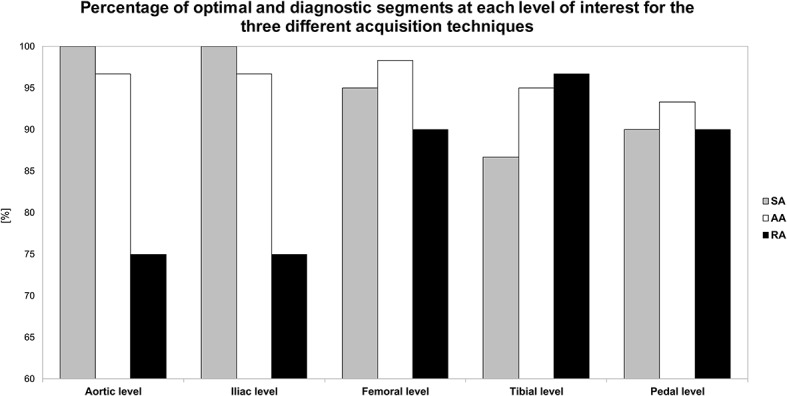
Bar graph represents the percentage of optimally enhanced segments on axial reconstructions for each of the three acquisition techniques. SA, standard acquisition technique; AA, adaptive acquisition technique; RA; retrograde acquisition technique.

Qualitative ratings of image quality at each level are presented in Table 5 and Figure 3. The Chi-square test showed a significant difference between the three techniques overall, with 4.7% of non-diagnostic segments for SA, 3.9% for AA, and 13.1% for RA (*p* < 0.01). In subgroup analysis including only the below-the-knee levels, there was no significant difference between the three acquisition techniques (*p* = 0.55); however, the percentage of non-diagnostic segments was lowest with AA compared to SA and RA (5.0, 7.8, 6.1% and, respectively). At the plantar level, percentages of optimal enhancement for SA, AA, and RA were 66, 83, and 66%, respectively.

**Table 5:** Number of optimal, non-optimal but diagnostic, and non-diagnostic segments at each level of interest for the three different acquisition techniques.

	**Level of interest**		**1**	**2**	**3**	**4**	**5**	**Total [%]**
Standard acquisition	O	Axial	20	20	17	13	14	84
		MIP	20	20	17	6	13	76
		VR	20	20	17	9	13	79
	NO	Axial	0	0	2	7	6	15
		MIP	0	0	2	9	3	14
		VR	0	0	2	8	5	15
	ND	Axial	0	0	1	0	0	1
		MIP	0	0	1	5	4	10
		VR	0	0	1	3	2	6
Adaptive acquisition	O	Axial	19	19	18	16	18	90
		MIP	18	18	17	15	16	84
		VR	19	19	18	14	16	86
	NO	Axial	1	1	2	4	2	10
		MIP	1	1	3	3	2	10
		VR	0	0	1	5	2	8
	ND	Axial	0	0	0	0	0	0
		MIP	1	1	0	2	2	6
		VR	1	1	1	1	2	6
Retrograde acquisition	O	Axial	6	7	16	17	14	60
		MIP	6	7	16	16	13	58
		VR	6	7	16	14	13	56
	NO	Axial	13	11	2	3	6	35
		MIP	6	6	2	3	3	20
		VR	8	7	2	5	5	27
	ND	Axial	1	2	2	0	0	5
		MIP	8	7	2	1	4	22
		VR	6	6	2	1	2	17

*N = 536 levels*.

*1, aortic level; 2, iliac level; 3, femoral level; 4, tibial level; 5, pedal level; O, optimal; NO, non-optimal but diagnostic; ND, non-diagnostic; MIP, maximum intensity projection; VR, volume rendering*.

**Figure 4:**
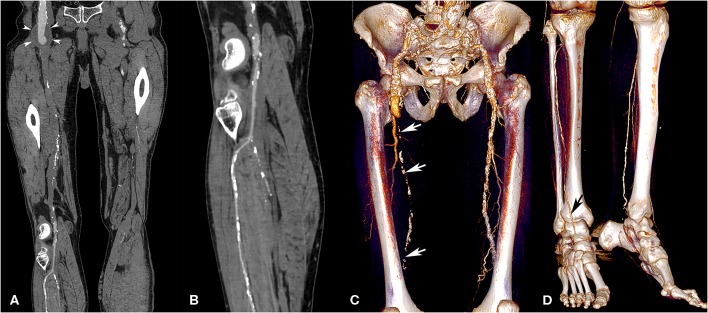
Lower extremity CT angiography obtained with the AA technique in a 75-year-old man with a history of bilateral chronic limb ischemia who had increasing pain on exertion for about a week. Curvilinear reconstructions of the right iliofemoral arterial axis **(A)** and anterior tibial artery **(B)** show optimal image quality despite the presence of an aneurysm of the abdominal aorta and right external iliac artery (arrowheads). Volume rendering reconstructions show a long-segment occlusion of the right superficial femoral artery (white arrows, **C**), yet an optimally enhanced anterior tibial artery (**D**, black arrow). Attenuation measurements were as follows: aortic level, 235 HU; iliac level, 222 HU (right) and 231 HU (left); femoral level, 188 HU (right), and 233 HU (left); tibial level 235 HU (right), and 218 HU (left); pedal level, 168 HU (right), and 130 (left). HU, Hounsfield units.

**Figure 5:**
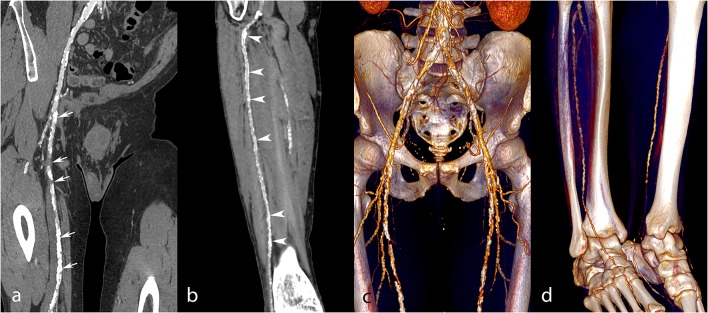
Lower extremity CT angiography obtained with the SA technique in a 54-year-old man with a history of insulin-requiring type 2 diabetes, hypertension, past stroke, and myocardial infarction, who was admitted due to Fontaine stage III peripheral arterial disease. Curvilinear reconstructions **(a,b)** and corresponding volume rendering reconstructions **(c,d)** of the right iliofemoral arterial system and anterior tibial artery. Several severe stenoses and occlusions were found along the superficial femoral artery (**a,c**, white arrows). At the tibial and pedal levels **(b,d)**, the arterial enhancement is non-optimal but sufficient for a diagnosis. Nevertheless, note the extensive arterial wall calcifications of the anterior tibial artery (white arrowheads) related to diabetes, rendering the analysis difficult. Attenuation measurements were as follows: aortic level, 261 HU; iliac level, 271 HU (right), and 256 HU (left); femoral level, 191 HU (right), and 176 HU (left); tibial level 187 HU (right), and 172 HU (left); pedal level, 112 HU (right), and 107 (left). HU, Hounsfield units.

**Figure 6:**
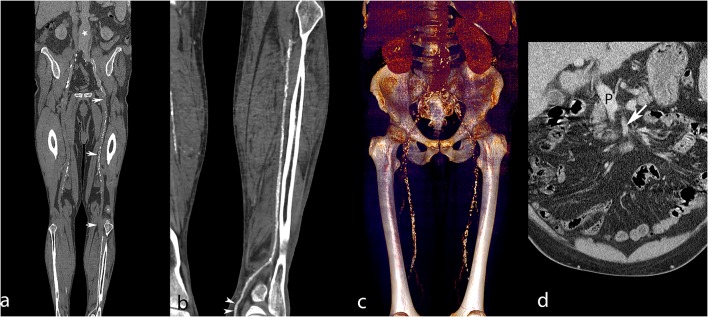
Lower extremity CT angiography obtained with the RA technique in a 65-year-old man with a history of ischemic and hypertensive heart disease, atrial fibrillation, chronic obstructive pulmonary obstruction, past stroke, and an aneurysm of the abdominal aorta. The patient was admitted for critical ischemia of the lower limbs, characterized by Fontaine stage IV disease of the left lower limb with superficial ulceration. Curvilinear reconstruction of the left aortopedal arterial system **(a)** shows inadequate enhancement of the abdominal aorta (white star) and absent enhancement of the superficial femoral artery due to an extended occlusion (white arrows). Curvilinear reconstruction of the left posterior tibial artery **(b)** shows optimal enhancement till the pedal level (white arrowheads). Volume rendering reconstruction of the abdominal aorta and femoral arteries **(c)** provides limited visualization of the arterial system and shows enhancement of the liver and kidneys related to venous contamination. Coronally reformatted slice through the abdomen **(d)** shows enhanced portal vein (P) and insufficiently enhanced superior mesenteric artery (white arrow), meaning that the sampling instant was too late for arterial phase imaging. Attenuation measurements were as follows: aortic level, 99 HU; iliac level, 85 HU (right) and 94 HU (left); femoral level, 123 HU (right), and 168 HU (left); tibial level 270 HU (right), and 305 HU (left); pedal level, 235 HU (right), and 273 (left). HU, Hounsfield units.

The effect of venous enhancement is presented in Table 6. The global influence of venous return on CTA interpretation was significantly different across the three acquisition techniques (*p* < 0.01). Venous contamination was particularly pronounced at the aortic level for in the RA group (9.4% of patients, as compared to 0% in the other groups). Figures 4–6 illustrate the clinical use of the AA, SA, and RA techniques.

**Table 6:** Number of segments with venous return and influence on the interpretation at each level of interest for the three different acquisition techniques.

	**Level of interest**		**1**	**2**	**3**	**4**	**5**	**Total [%]**
Standard acquisition	A	Axial	20	20	20	20	16	96
		MIP	20	20	20	20	19	99
		VR	20	20	20	19	19	98
	P-NI	Axial	0	0	0	0	4	4
		MIP	0	0	0	0	0	0
		VR	0	0	0	1	0	1
	P-I	Axial	0	0	0	0	0	0
		MIP	0	0	0	0	1	1
		VR	0	0	0	0	1	1
Adaptive acquisition	A	Axial	20	20	20	19	18	97
		MIP	20	20	20	19	20	99
		VR	20	20	20	19	18	97
	P-NI	Axial	0	0	0	1	2	3
		MIP	0	0	0	1	0	1
		VR	0	0	0	1	2	3
	P-I	Axial	0	0	0	0	0	0
		MIP	0	0	0	0	0	0
		VR	0	0	0	0	0	0
Retrograde acquisition	A	Axial	13	16	20	20	19	88
		MIP	17	18	20	20	19	94
		VR	10	12	20	20	19	81
	P-NI	Axial	7	4	0	0	1	12
		MIP	3	2	0	0	1	6
		VR	6	6	0	0	1	13
	P-I	Axial	0	0	0	0	0	0
		MIP	0	0	0	0	0	0
		VR	4	2	0	0	0	6

*N = 536 levels*.

*1, aortic level; 2, iliac level; 3, femoral level; 4, tibial level; 5, pedal level; A, venous return absent, P-NI, venous return present with no influence; P-I, venous return present with influence on the interpretation; MIP, maximum intensity projection; VR, volume rendering*.

## Discussion

There are various CT angiography techniques to assess the arterial system of the lower limbs (10). One may vary the gantry rotation time or table feed to speed up or slow down the acquisition. One may also adapt the contrast medium injection protocols or even change the direction of acquisition (anterograde or retrograde).

The AA technique has shown to be robust (4, 11, 12), making it possible to “synchronize” the angiographic acquisition to the patient's circulation status. Accordingly, the risk to outrun the contrast medium bolus or to have venous contamination is minimized. Such advantages are particularly valuable in patients with disturbed arterial circulation. AA acquisition provides solid sensitivity and specificity (4). In the aortoiliac arteries, the sensitivity and specificity were reported to be 100%, and in the femoropopliteal and the below-the-knee arteries, the sensitivity and specificity remained high (between 91 and 95%). The reliability of the AA technique is well-reflected in our study, based on the high rate of optimal attenuation, especially in the plantar segments, and by the absence of severe venous contamination. The attenuation homogeneity analysis also supports the AA technique, which provided the lowest difference of attenuation of the three acquisition methods. Therefore, adapting the speed of the table feed allows optimal availability of the contrast medium along the arterial tree of the lower extremities. Our results confirmed that AA is the method providing the best performance in terms of arterial enhancement, from the abdominal aorta to the plantar level, both in quantitative and qualitative image quality assessment. Of course, some differences with other techniques were not statistically significant. However, it is interesting to notice that the AA group included two of the three patients referred to CTA for aneurysmal disease, two of the three patients with heart failure, 55% of identified peripheral aneurysms, and 35% of limbs with relevant stenoses. A drawback of the AA technique is the need for an accurate calculation of the aortopopliteal transit time. Although this calculation is not particularly complicated, it still requires some training for the technologists and supervision from the radiologists. It may be time-consuming and a learning curve is involved. Another drawback of AA is the need to use a test bolus, which adds to the contrast medium bolus used for the angiographic acquisition. Additional contrast medium administration should be avoided in PAD patients suffering from kidney failure.

The SA technique is well-established (10). Sensitivity and specificity have been widely studied and have proved to be very high (2, 5, 6, 8, 13–15). In the above-the-knee and below-the-knee levels altogether, the sensitivity and specificity vary between 83 and 99% for the detection of stenosis >50% in lumen diameter reduction. Considering the below-the-knee levels only, sensitivity and specificity remain high: between 74 and 100%, although sensitivity seems to be somewhat lower. SA is convenient to use and feasible on all the currently available CT systems. However, it lacks the customizable feature of the AA method. This might not be disadvantageous in patients with chronic Fontaine stage II disease, but may be detrimental in patients with diabetes (16) or acute limb ischemia (17), since distal vessels are often much more calcified in diabetic patients and acute occlusions are likely to restrict contrast medium inflow into the distal vessels. While we do not find a statistically significant difference between the three techniques for the below-the-knee levels in the overall population, SA showed a slightly inferior performance compared to AA both in terms of attenuation and qualitative ratings. Despite this, SA is still a valuable alternative for patients with impaired kidney function, since the additional contrast agent injection for the test bolus can be avoided.

The RA method is a more recent technique to perform CT angiography of the lower extremities. While caudocranial acquisition has long been recommended for CT venography (18), scarce data is available regarding arterial run-off CT angiography using caudocranial acquisition. The justification for this method is to maximize the enhancement of the distal vessels. As already mentioned, in patients with critical lower limb ischemia, the distal vascular segments are frequently challenging to evaluate. The RA technique is only feasible on the last generations of scanners, as it requires fast table feed. Under the assumption that such a fast acquisition speed may obviate the problem of venous contamination, superior image quality of the distal vessels should be obtained. In our population, the data did not demonstrate a clear advantage of the RA method. Nevertheless, attenuation values were slightly higher at the tibial and pedal level with RA, as compared to SA and AA. That is, RA may have some advantages in patients with chronic limb ischemia, where detailed information regarding the distal arterial tree is required to support the decision-making process and help establish the best strategy for endovascular revascularization, including vascular access and target lesions to treat. Decreased attenuation values for more proximal levels are likely related to the acquisition time on our CT system, which probably was too long compared to the transit time of contrast material from the abdominal aorta to the feet. We can expect to overcome this limitation with more recent CT technology (256-MDCT and more) shortly. A recent study conducted using a 196-MDCT system compared fast RA with standard speed craniocaudal acquisition and demonstrated not only better intraluminal attenuation with RA, but also radiation dose reduction in the order of 40% (19).

When stratifying limbs by the presence or absence of significant stenosis and the presence or absence of aneurysms, more information regarding the behavior of the bolus may be extracted and accounted for, especially with the two anterograde techniques. For example, the presence of stenosis did not seem to hinder the opacification across the three different acquisition methods, which might be linked to the fact that patients develop sufficient collateral reserve to allow the contrast medium bolus to reach the distal vessels. On the other hand, our results showed significant attenuation differences in the proximal levels in the presence of aneurysmal disease (most aneurysms in our population were located in the iliac arteries), and when scanning with AA and RA. Interestingly, the attenuation was substantially higher in the presence of aneurysmal disease with AA and substantially lower with RA, which confirms the robustness of AA and likely results from contrast medium pooling in the aneurysmal segment. As the sampling of the proximal levels is performed early with AA and late with RA, an inverse effect is observed between these two techniques. With SA, this effect is limited, which can be explained by the fact that SA provides its peak attenuation in the proximal vessels (Figure 2).

We used the same contrast agent concentration, volume, and injection rate for all groups, in order to limit biases related to the variation of contrast medium injection schemes. We did not assess the effect of patients' body weight on contrast medium attenuation, which can influence the arterial enhancement (20). It is known that larger patients have a higher cardiac output and increased central blood volume, which could influence vascular enhancement (21–23). Still, we found no significant difference in body weight between the three groups, and we do not think that the body weight factor may have a relevant influence on the results of our study. Moreover, in a recent study by Horehledova et al. (12), AA helped reduce the volume of contrast medium used for lower extremity CTA to 45 mL, of which 15 mL were for the test bolus, irrespective of body weight. Besides, neither the age nor the sex ratio was different between the groups. This observation can also be applied to the presence of heart failure. One further limitation of the study is the addition of 30 mL of contrast medium for the test bolus in the AA group. Additional iodinated contrast medium volume may theoretically increase the measured attenuation. However, the acquisition takes place several minutes after the injection of the test bolus, due to the time needed to compute the aortopopliteal transit time. We believe the effect is limited. Moreover, our results did not show extra venous return with AA compared to the other methods, which demonstrates the negligible influence of the bolus test. Finally, by study design, each patient had one acquisition technique which might induce potential biases linked to uneven distribution of anatomical and pathological (occlusive or aneurysmal disease) features in the three groups.

## Conclusion

Considering the attenuation values and their homogeneity, AA performed the best, and also provided the highest rate of optimal vascular enhancement. In centers with trained technologists and radiologists, AA should be considered as the acquisition method of choice, specifically in patients with critical limb ischemia and/or aneurysmal diseases. SA might represent an alternative option in patients with impaired kidney function, as the additional contrast medium of the test bolus can be avoided. Finally, despite its less optimal performance for the more proximal levels, the RA technique may be useful in occlusive disease affecting the below-the-knee arteries. Moreover, future developments of CT technology and the increasing availability of higher acquisition speeds will likely contribute to overcoming the major limitations of RA.

## Data Availability Statement

The datasets generated for this study are available on request to the corresponding author.

## Ethics Statement

Written informed consent was obtained from every patient participating and the Ethics Committee of the Canton de Vaud approved the study.

## Author Contributions

All authors contributed in drafting the manuscript and revising it critically. Furthermore, they were involved in the following tasks. DR: data analysis and interpretation, literature review. T-LL: data acquisition, analysis, and interpretation, literature review. AK: data acquisition and analysis. P-MM-V: data analysis, statistical analysis. GF: data acquisition and analysis. SQ: study design, data analysis, and interpretation, literature review.

## Conflict of Interest

The authors declare that the research was conducted in the absence of any commercial or financial relationships that could be construed as a potential conflict of interest.
